# Diagnostic Accuracy of Five Different Fecal Markers for the Detection of Precancerous and Cancerous Lesions of the Colorectum

**DOI:** 10.1155/2016/2492081

**Published:** 2016-06-16

**Authors:** Mariann Rutka, Renáta Bor, Anita Bálint, Anna Fábián, Ágnes Milassin, Ferenc Nagy, Zoltán Szepes, Mónika Szűcs, László Tiszlavicz, Klaudia Farkas, Tamás Molnár

**Affiliations:** ^1^First Department of Medicine, University of Szeged, Korányi Fasor 8-10, Szeged 6720, Hungary; ^2^Department of Medical Physics and Informatics, University of Szeged, Korányi Fasor 9, Szeged 6720, Hungary; ^3^Department of Pathology, University of Szeged, Állomás Utca 2, Szeged 6720, Hungary

## Abstract

*Background.* Colorectal cancer (CRC) is the second deadliest malignancy worldwide. This study aimed to compare the diagnostic accuracy of different fecal markers in the detection of colorectal adenomas and cancer.* Methods.* Stool samples of patients referred to colonoscopy were collected for the analysis of tumor M_2_ pyruvate kinase (M_2_PK), human hemoglobin (Hb), hemoglobin/haptoglobin (Hb/Hp) complex, fecal calprotectin (FC), and matrix metalloproteinase-9 (MMP-9).* Results.* Sensitivity and specificity of M_2_PK for adenomas sized > 1 cm were 60% and 67.5% and for CRC were 94.7% and 67.5%. Sensitivity and specificity of iFOBT for adenomas sized ≥ 1 cm were 80% and 72.5% and for CRC were 94.7% and 72.5%. Sensitivity and specificity of Hb/Hp complex for adenomas sized ≥ 1 cm were 80% and 52.9% and for CRC were 100% and 52.9%. Sensitivity of FC and MMP-9 for CRC was 77.8% and 72.2%. Combined use of M_2_PK, iFOBT, and FC resulted in a sensitivity and specificity of 95% and 47.5% for the detection of adenomas sized ≥ 1 cm.* Discussion.* In CRC, sensitivity of M_2_PK, iFOBT, and Hb/Hp complex proved to be high. Combined use of M_2_PK, iFOBT, and FC may be valuable in the detection of large adenomas.

## 1. Introduction

Colorectal cancer (CRC) incidence and mortality rates vary markedly worldwide. Globally, CRC is a third most common cancer, being a significant leading cause of cancer death in both genders [1]. Furthermore, the incidence of CRC is increasing in Central European countries [1]. The Hungarian mortality rates for CRC proved to be the highest among men in Europe in 2012 [2].

The vast majority of CRC cases are sporadic colon cancers characterized by a multistep carcinogenic process [3]. Advanced adenomas greater than 10 mm in diameter with high-grade dysplasia or with more than 20% villous component are considered to be the clinically relevant precursors of CRC. However, the long premalignant phase of sporadic CRCs provides a good opportunity for successful screening and intervention.

Colonoscopy is considered the gold standard of CRC screening tools. However, mainly due to the invasive nature of colonoscopy, the acceptance of this type of screening method among patients is low. The most commonly used noninvasive screening method for CRC is the guaiac fecal occult blood test (gFOBT) based on the detection of hemoglobin peroxidase activity in the stool. However, the sensitivity and the specificity of this test are not good enough to safely rule out the presence of CRC or adenomas which is why there is a great need for a better noninvasive marker for these conditions. In the case of proximal malignant lesions, hemoglobin/haptoglobin (Hb/Hp) detection can be superior to Hb detection alone since Hb/Hp complex remains stable over the entire course of the large bowel in comparison to Hb degraded on the way [4–6]. M_2_ pyruvate kinase (PK) is a biochemical form of PK which is a key enzyme in cancer cell metabolism [7]. M_2_PK is expressed in normal proliferating cells, embryonic cells, adult stem cells, and cancer cells [8]. Elevated levels of M_2_PK have been detected in colonic adenocarcinoma [9]. Calprotectin is a calcium-binding and zinc-binding protein complex that is abundant in the cytosol of inflammatory cells [10, 11]. Fecal calprotectin (FC), a biomarker of intestinal inflammation, has been in clinical use for years in inflammatory bowel disease [11–13]. FC has been shown to be elevated in CRC and has been suggested to be for screening high risk groups for CRC [14].

Matrix metalloproteinase (MMP) is a large family of calcium-dependent zinc-containing endopeptidases responsible for tissue remodelling and degradation of the extracellular matrix components, including collagens, elastins, gelatin, matrix glycoproteins, and proteoglycan, in multiple disease settings including malignant processes. MMP-9 subtypes are believed to play a crucial role in the progression and metastasis formation of many tumors, including CRC [15].

Since the majority of the abovementioned tests are not officially recommended in the CRC screening guidelines and some of them have not been tested previously, the aim of this study was to compare the diagnostic accuracy of different fecal markers in the detection of precancerous and cancerous lesions of the colorectum and to find the most accurate for CRC screening.

## 2. Methods

### 2.1. Patient Population and Study Protocol

Patients from the 1st Department of Medicine, University of Szeged, who were referred for colonoscopy were invited to participate in the study. Data on symptoms, smoking habits, family history, and current medication were collected. Every patient was informed about the study details and asked to sign written consent. The patients were instructed for sample collection and handling. All patients were asked to collect stool samples one day before administration of bowel preparation. Plastic containers were provided for feces collection. After bringing the samples at the lab of the clinic, they were frozen at −20°C until further analysis. Patients did not have to keep a special diet and were told to take their usual medications. Selection of the patient groups with adenomas sized <1 cm and ≥1 cm and CRC was based on the endoscopic and histological finding. The stool testing for M_2_PK, iFOBT, FC, and MMP-9 was carried out by a single trained person who was blinded to the results of the colonoscopy.

The study was approved by the Regional and Institutional Human Medical Biological Research Ethics Committee of the University of Szeged.

### 2.2. Measurement of Fecal M_2_PK and iFOBT

A combined rapid immunochromatographic lateral flow test was used for simultaneous detection of enzyme biomarker M_2_PK and human hemoglobin (combined M_2_PK and HB, 2 in 1 Quick Test, ScheBo® Biotech). For these measurements, stool samples were thawed and a special stick capturing 4 mg of stool was loaded. These tests are based on visual inspection of colors at test and control lines. The result is exclusively qualitative (detection limit of M_2_PK was 4 U/mL; detection limit of Hb was 15 ng/mL).

### 2.3. Measurement of Fecal Hb and Hb/Hp Complex

Hb/Hp complex was determined from stool samples with a visual immunochromatographic quick test: ColonView Hb and Hb/Hp fecal occult blood test (Biohit HealthCare; detection limit of Hb was 15 ng/mL; detection limit of Hb/Hp was 4 ng/mL).

### 2.4. Measurement of FC and Fecal MMP-9

For FC measurements, fecal specimens were thawed at 4°C. FC level was quantified by using enzyme-linked immunosorbent assay (Quantum Blue, BÜHLMANN Laboratories Ltd., Schönenbuch) according to the manufacturer's instructions. For MMP-9 measurements, 1 g of fecal samples was diluted, mixed, homogenised in 4 mL of ice-cold Tris-buffer (0.15 M NaCl + 20 mM Tris-HCl, pH 8.3), and then centrifuged. MMP-9 was also measured by quantitative enzyme-linked immunosorbent assay (R&D Systems, Abingdon, UK) [16].

### 2.5. Colonoscopy and Histological Examination

Diagnosis was based on the endoscopic and histopathological findings. Colonoscopies were performed by three experienced endoscopists (TM, ZSZ, and FN) who were blinded to fecal tests results. Carcinomas were classified according to the Dukes staging system and location. Adenomatous polyps were classified according to histopathological characteristics, size (large polyps: ≥1 cm; small polyps: <1 cm), and location. All colonoscopy biopsies were examined by an expert pathologist (LT). The diagnoses were reported using the standard WHO classification of colorectal neoplasia. In addition to their size, all polypoid lesions were classified as hyperplastic polyps or adenomas, being further classified according to their histological pattern as tubular, tubulovillous, villous, or serrate adenomas.

### 2.6. Statistical Analysis

CRCs and adenomas were analysed separately. The diagnostic value of fecal markers for detecting adenomas and CRCs was assessed by calculating the sensitivity and the specificity of the test. Correlations between FC and MMP-9 and endoscopic findings were determined by ANOVA method. The cut-off levels, specificity, and sensitivity between CRC and control groups were calculated using the receiver operating characteristic (ROC) analysis. All statistical analyses were carried out using STATA 9 (StataCorp, TX, 2005).* P* values < 0.05 were considered to be statistically significant.

## 3. Results

### 3.1. Patient Population

Ninety-five consecutive in- and outpatients admitted for total colonoscopy between September 2014 and April 2015 were prospectively enrolled in the study. Indications for colonoscopies were abdominal complaints, bloody stool, family history of CRC, and prior colorectal adenoma. Patients with active gastrointestinal bleeding, menstruation, and past history of total colectomy were excluded from the study. Study groups were defined on the basis of the result of colonoscopy and histological evaluation.

Mean age was 67 years (range: 21–92) in study population. 57 female and 38 male patients were in these three groups, respectively. Demographic characteristics of the study population are summarized in Table 1. Family history of CRC was reported by 26 patients. Considering therapy, 26 patients received aspirin or clopidogrel and 4 received acenocoumarol or heparin at the time of the investigation.

### 3.2. Colonoscopic and Histological Findings

Forty of the 95 patients included in the study represented the control group without any premalignant or malignant findings on endoscopy. Nine of the control patients presented with initial diverticulosis without any sign of inflammation. Colonoscopic findings in the remaining patients of the control group were totally normal.

Thirty-six patients were diagnosed with adenomas (adenoma group). In the adenoma group, 16 patients presented with adenomas sized <1 cm and 20 with adenomas sized ≥1 cm. Adenomas sized <1 cm were equally located at the proximal and the distal part of the colon. The location of adenomas sized ≥1 cm in the majority (65%) of the patients was the proximal part of the colon. In twenty-three adenomatous cases, a histologic sample was obtained. In the remaining thirteen cases, the samples were less than 1 cm and did not suggest the presence of malignancy. Based on the histological assessment of the samples (*n* = 23), in 78.3% of the cases (in 18 patients), the adenomas were with low-grade dysplasia; in 13% (in 3 patients), adenomas were with high-grade dysplasia; and in 8.7% (in 2 patients) there were hyperplastic polyps. In 56.5% of the patients the adenomas were of the tubular type, in 4.3% they were of the villous type, and in 30.4% they belong to the tubulovillous type.

Cancer was found in 19 cases, and, according to their histological evaluation, the tumors were identified as adenocarcinomas. In 89% of the patients, the cancer was located in the distal colon (in 10 patients in the rectum and in 7 patients in the sigmoid colon). In the remaining 2 cases, the tumor was located in the distal part of the transverse colon. 28.8% of these patients had a family history of CRC. The numbers of patients having different stages of cancer according to Dukes classification are shown in Table 2.

### 3.3. Diagnostic Accuracy of Fecal Markers in Adenomas and CRCs

M_2_PK was positive in 32.5% of the patients with normal colonoscopy, in 43.7% with adenomas sized <1 cm, in 60% with adenomas sized ≥1 cm, and in 94.7% with CRCs. M_2_PK sensitivity for adenomas sized >1 cm was 60%, and specificity was 67.5%. Sensitivity and specificity for CRC were 94.7% and 67.5%. Sensitivity and specificity for iFOBT for adenomas sized ≥1 cm were 80% and 72.5% and for CRC were 94.7% and 72.5%. The Hb/Hp (Hb and Hb/Hp ColonView Biohit test) complex was positive in 47.1% of the patients with normal colonoscopy, in 50% with hyperplastic polyps, in 54% with adenomas sized <1 cm, in 80% with adenomas sized ≥1 cm, and in 100% with CRC. Sensitivity and specificity of Hb/Hp complex for adenomas sized ≥1 cm were 80% and 52.9% and for CRC were 100% and 52.9%.

FC and MMP-9 differed significantly between the control and CRC group (*p* = 0.022; *p* < 0.001); however, no difference was found in FC and MMP-9 concentrations between the control and the adenoma groups. FC was significantly lower in adenomas sized <1 cm compared to CRCs but did not differ when compared to adenomas sized ≥1 cm with CRCs (*p* = 0.022, *p* = 0.089). MMP-9 proved to be significantly lower compared to either adenomas sized <1 cm with CRCs or adenomas sized ≥1 cm with CRCs (*p* ≤ 0.001 and *p* ≤ 0.001).

Sensitivity of FC for CRC was 77.8%, while specificity for CRC was 70%. The cut-off value of FC for the detection of CRC was 128.5 *μ*g/g (AUC = 0.77, *p* = 0.001). Sensitivity of MMP-9 for CRC was 72.2%, while specificity was 95%. The cut-off value of MMP-9 for the detection of CRC was 1.12 ng/g (AUC = 0.77, *p* < 0.001).

Using combinations of fecal markers, the highest sensitivity for detection of adenomas sized ≥1 cm was revealed when combining M_2_PK, iFOBT, and FC (with the cut-off of 128.5 *μ*g/g) resulting in a sensitivity and specificity of 95% and 47.5% for the detection of adenomas sized ≥1 cm.

Sensitivities, specificities, and positive and negative predictive values of the fecal markers are summarized in Table 3.

We did not find any relationship between platelet aggregation inhibitor therapy and positive results of the different hemoglobin tests (logistic regression: Hb_ScheBo_ *p* = 0.4; Hb/Hp_Biohit_ *p* = 0.609).

## 4. Discussion

CRC is a major health problem worldwide. Despite being a good candidate for screening due to its detectable premalignant lesions, mortality rates of CRC are still significant in Hungary [17]. Early detection by an accurate, noninvasive, cost-effective, simple-to-use screening technique is central to decrease the incidence and mortality of this disease. Patient discomfort, invasiveness, embarrassment, high cost, and considerable expertise and equipment required for the procedure may all limit the appeal of this screening technique and the increasing number of examinations puts a huge burden on the gastroenterologists. Thus, there is still an unmet need for suitable noninvasive biomarkers to screen for CRC.

In this prospective colonoscopy-controlled study, we assessed the sensitivity, specificity, and positive and negative predictive values of different noninvasive fecal markers for the detection of adenomas and CRC. For adenomas sized ≥1 cm, iFOBT showed the highest sensitivity and M_2_PK the highest specificity. For CRC, M_2_PK and Hb/Hp complex showed the highest sensitivity and fecal MMP-9 the highest specificity. FC and fecal MMP-9 concentrations did not differ between the control and the adenoma group, although they proved to be beneficial mainly in the detection of adenomas sized ≥1 cm and CRC. In CRCs, the sensitivities of FC and MMP-9 were 78% and 72%, with specificities of 70% and 95%. The combination of M_2_PK, iFOBT, and FC increased their sensitivity for the detection of adenomas sized ≥1 cm up to 95%.

The study has some limitations. First, we collected stool samples before performing colonoscopy; thus, we were blinded to the findings and the number of high-grade adenomas finally proved to be low. We do not know whether there would be associations between adenomas and fecal markers if the number of adenomas with high-grade dysplasia would be higher. Second, M_2_PK and Hb tests and the Hb/Hp complex were all qualitative tests based on a chromatographic method interpreted visually which may limit their assessment in case of borderline results. Therefore, it may be difficult to compare the results with those of FC and MMP-9. However, these tests are simple, do not require specific laboratory equipment, and therefore are less expensive than the quantitative methods.

The guaiac-based FOBT (gFOBT) is the oldest and most commonly used noninvasive test for detecting CRC [18, 19]. Although the test is relatively inexpensive and easy to perform, false-positive and false-negative results compose its main limitation resulting in limited sensitivity for detecting cancer and advanced adenomas [20]. The Hb/Hp complex shows higher stability against degradation than Hb itself. Sieg et al. revealed that Hb/Hp complex has a comparable sensitivity to fecal Hb for CRCs (87% for both) and higher sensitivity for adenomas (76% versus 54%) [4]. However, these tests are based on the bleeding property of the adenomas. Since early-stage cancers or advanced adenomas are unlikely to bleed continuously, 100% of clinical sensitivity cannot be achieved with the use of these tests. That is why the identification of novel fecal-based biomarkers is important.

M_2_PK is expressed by proliferating cells, in particular the tumor cells being direct target of several oncoproteins. Among the first studies assessing the sensitivity of M_2_PK for the detection of CRC, Shastri et al. revealed that fecal M_2_PK assay had sensitivity and specificity of 81.1 and 71.1% for diagnosing CRC at a cut-off value of 4 U/mL whereas FOBT showed a sensitivity of 36.5% and specificity of 92.2% for CRC. They concluded that M_2_PK is a poor screening biomarker, due to its low specificity [21]. However, a meta-analysis including 17 studies performed between 2006 and 2010 found the mean fecal M_2_PK sensitivity and specificity to be 80.3% and 95.2% for CRC and a sensitivity of 44% for adenomas >1 cm [22].

According to our results, M_2_PK, Hb, and Hb/Hp tests show better sensitivity in the detection of CRC than advanced adenomas. The study by Kim et al. revealed that the sensitivity of iM_2_PK, an immunochromatographic qualitative method for fecal M_2_PK for CRC, was 92.8% and for adenomatous lesions the sensitivity was 69.4% [23]. Compared with M_2_PK ELISA, iM_2_PK exhibited significantly enhanced sensitivity for CRC (97.5% versus 80%, *p* = 0.03).

FC is valuable in differentiating functional and organic bowel diseases. FC was shown to be more sensitive (79%) but less specific (72%) for CRC and adenomatous polyps as a combined group than gFOBT [24]. MMP-9 is an important member of the gelatinases involved in the development of several human malignancies [25]. Yang et al. found that MMP-9 expression in colon cancer tissues was significantly higher than that in corresponding distal normal mucosa tissue [15]. However, the sensitivity of MMP-9 detected in feces has not been examined previously. Our results revealed a moderate sensitivity of 72% and a good specificity of 95% for fecal MMP-9 in CRC. However, neither FC nor fecal MMP-9 provided valuable information on the detection of adenomas.

In this study, we compared the sensitivity and specificity of several fecal markers for the detection of colorectal cancers. The strengths of this study are the design that allowed directly calculating sensitivity and specificity of the different fecal markers, since every patient underwent colonoscopy after stool sample collection. This was the first time when five biomarkers were simultaneously studied. Fecal M_2_PK has the advantage that it detects both bleeding and nonbleeding tumors and adenoma. Conversely, fecal M_2_PK does not have false-positive results due to various noncancerous sources of bleeding. Furthermore, FC, MMP-9, and fecal M_2_PK are also sensitive to intestinal inflammation (inflammatory bowel disease, diverticulitis) increasing the proportion of false-positive cases. In this study, we performed examinations for patients with GI symptom(s) not as a part of screening process because by this method we could disclose false-positive results and could determine specificity data as well.

In our cohort, the highest sensitivity and specificity were achieved by the use of combined M_2_PK and iFOBT test in the detection of CRC. FC seems to be a useful adjuvant to the investigation of patients at high risk for colorectal neoplasia, while fecal MMP-9 may be a promising factor for detection of CRC. Although, in CRC, sensitivity of M_2_PK, iFOBT, and Hb/Hp complex proved to be high, in adenomas sized ≥1 cm, sensitivity decreased significantly. Therefore, none of these markers are unique for detection of precancerous lesions of the colorectum. However, our result revealed that combined use of M_2_PK, iFOBT, and FC may be valuable in the detection of large adenomas.

We recommend these noninvasive fecal tests in low-risk patients and in patients who do not have comorbidities. Results of FOBT may be false positive if the source of bleeding is not an adenoma or a malignant disease (diverticulitis, hemorrhoids, and anticoagulant therapy). However, inflammatory diseases of the colon (diverticulitis, different infections, and inflammatory bowel diseases) and extraintestinal cancer (cancer in the hepatobiliary tract, pancreas) or inflammation (hepatitis) may affect the results of the inflammatory marker test; thus, in these cases, we recommend colonoscopy as a one-step investigation. High-risk patients (who had at least one relative with early CRC or adenoma or had at least two relatives with CRC or adenoma) with symptoms or patients who have early (under the age of 60) CRC or adenoma among their relatives should also undergo colonoscopy. However, it is not questionable whether continued efforts are needed to discover effective tests to identify patients with nonhereditary risk factors and to develop invasive and cost-effective screening modalities.

## Figures and Tables

**Table 1:** Demographic characteristics of the study population.

Demographic data	All patients (95)	Control group (40)	Adenoma group (36)	Cancer group (19)
*Female/male*	38/57	19/21	14/22	5/14
*Age (years)*	67 (21–92)	67 (21–87)	68 (51–81)	65 (44–92)
*Current smokers*	13 (13.7%)	4 (10%)	5 (13.9)	4 (21.1)
*Comorbidities*				
Hypertension	54 (56.8%)	23 (57.5%)	22 (61.1%)	9 (47.4%)
Diabetes mellitus	21 (22.1%)	7 (17.5%)	8 (22.2%)	6 (31.6%)
Hyperlipidaemia/hypercholesterinemia	22 (23.2%)	9 (22.5%)	11 (30.6%)	2 (10.5%)
Cardiovascular disease	25 (26.3%)	11 (27.5%)	10 (27.7%)	4 (21.1%)
Cerebrovascular disease	13 (13.7%)	6 (15%)	4 (11.2%)	3 (15.8%)
Hyper/hypothyroidism	13 (13.7%)	5 (12.5%)	7 (19.4%)	1 (5.3%)
Pulmonary disease	6 (6.3%)	4 (10%)	2 (5.6%)	0
Gout	11 (11.6%)	5 (12.5%)	5 (13.9%)	3 (15.8%)
Autoimmune disease	4 (4.2%)	0	3 (8.3%)	1 (5.3%)
Malignant disease (simultaneously)	3 (3.2%)	1 (2.5%)	2 (5.6%)	0
Hepatitis (B, C)	2 (2.1%)	1 (2.5%)	1 (2.8%)	0
Diverticulum	24 (25.3%)	11 (27.5%)	10 (27.8%)	3 (15.8%)
Haemorrhoids	20 (21.1%)	11 (27.5%)	7 (19.4%)	2 (10.5%)

**Table 2:** The numbers of patients having different stages of cancer according to Dukes classification.

Dukes stage	Patients
Carcinoma in situ	1
Dukes A	3
Dukes B	9
Dukes C	1
Dukes D	5

**Table 3:** Sensitivities, specificities, and positive and negative predictive values of the fecal markers.

Parameters	M_2_-PK_ScheBo_	HB_SchBo_	HB/HP_biohit_	Calprotectin	MMP-9
*Sensitivity*					
Adenoma sized ≥1 cm	60	80	80.0		
CRC	94.7	94.7	100.0	77.8	72.2
Adenoma sized ≥1 cm + CRC	76.9	87.2	90.9		

*Specificity*					
Adenoma sized ≥1 cm	67.5	72.5	52.9		
CRC	67.5	72.5	52.9	70.0	95.0
Adenoma sized ≥1 cm + CRC	67.5	72.5	52.9		

*PPV* (%)					
Adenoma sized ≥1 cm	80	59.2	42.9		
CRC	85.7	62	52.9	53.8	86.6
Adenoma sized ≥1 cm + CRC	69.7	75.5	65.2		

*NPV* (%)					
Adenoma sized ≥1 cm	77.1	96.6	85.7		
CRC	96.4	96.6	100.0	87.5	88.3
Adenoma sized ≥1 cm + CRC	75	85.3	85.7		
